# Cultural barriers and facilitators of the parents for human papillomavirus (HPV) vaccination uptake by their daughters: A systematic review

**DOI:** 10.1016/j.jped.2024.07.012

**Published:** 2024-11-04

**Authors:** Noor Shuhada Salleh, Khatijah Lim Abdullah, Heng Yee Chow

**Affiliations:** a Universiti Sains Malaysia, School of Health Sciences, Nursing Program, Kelantan, Malaysia; b Sunway University, School of Medical and Life Sciences, Department of Nursing, Bandar Sunway, Selangor, Malaysia; c Universiti Kebangsaan Malaysia, Faculty of Medicine, Department of Nursing, Cheras, Kuala Lumpur, Malaysia; d University College London, UCL Great Ormond Street Institute of Child Health, London, United Kingdom

**Keywords:** Papillomavirus vaccines, Vaccination hesitancy, Parents, Culture, Systematic review

## Abstract

**Objective:**

There is a pressing need for public health practitioners to understand cultural values influencing parents on the uptake of human papillomavirus (HPV) vaccination for their daughters, which is presenting a growing challenge to close the immunization gap worldwide. Parental decisions were predominantly shaped by cultural norms and values. This systematic review encompasses parental perspectives on the influence of cultural values on the uptake of HPV vaccination by their daughters.

**Method:**

This systematic review was registered on PROSPERO CRD42020211324. Eligible articles were selected from CINAHL, PsycINFO, EMBASE, PubMed and Science Direct. Original qualitative studies exploring parental perspectives on the influence of cultural values on the uptake of HPV vaccination by their daughters under the age of 18, published in the English language with no restriction dates were reviewed. Two authors independently screened abstracts, conducted the fill-text review, extracted information using a standardized form, and assessed study quality. A third author is needed to resolve the disagreements if necessary.

**Results:**

Of the 1552 citations, 22 were included, with information on 639 parents. Five themes emerged from the data: sexuality-related concerns; upbringing and moral values; obligation to protect; external influences; and vaccine-related concerns.

**Conclusion:**

This systematic review is beneficial to identify and understand the culturally related facilitators and barriers to HPV vaccination among young women for the development of strategies to optimize HPV vaccine coverage among this population group by the policymakers.

## Introduction

Vaccinations are effective in treating illness and preventing death from various vaccine-preventable diseases. Specifically, the human papillomavirus (HPV) vaccine is a significant advancement in reducing women's risk of cervical cancer.^1^ The World Health Organization's (WHO) new guidelines, published in 2020, recommend 2 doses of HPV vaccination to be administered among girls aged 9 to 14 years before they become sexually active.^2^ However, the promotion of the uptake of HPV vaccination remains a challenging task for pediatricians and vaccination hesitancy remains one of the most important barriers to succeeding public health campaigns against (HPV). This vaccination hesitancy may include components of trust, convenience, complacency, communication, and context.^2^

Parents are assumed to be the people with the greatest influence and responsibility when it comes to the prioritization of their children's best interests, especially for younger children with low levels of maturity to understand the principal benefits and risks of a decision. According to the laws and regulations in place in most countries, individuals above the age of 18 years are allowed to give consent but some countries have fixed the age of consent specifically to allow HPV vaccination at age 12.^3^ However, in most countries, parents/legal guardians have overall authority on this issue.^3^ Therefore, understanding parental acceptance of HPV vaccination is essential as governments consider how such vaccine promotion programs should be implemented.

It appears that cultural values such as religion, beliefs, and language need to be meticulously considered, as they could prove a powerful indicator in the uptake of HPV vaccination.^4^, ^5^, ^6^, ^7^ A cultural value is an emotionally charged concept, a recognized standard, or a core belief, that serves as a rule and forceful goal to direct people's thoughts, perceptions, and behavior.^8^ For this review, the scope of the cultural values is regarded as any factors related to parental culture that influenced intention, experiences, thoughts, views, beliefs, perceptions, feelings, and opinions demonstrated by parents towards HPV vaccination for their daughters.

To our knowledge, several systematic reviews looked at the various issues related to HPV vaccination, however, fewer reviews were identified that focused specifically on the influence of cultural values. These studies were published from 2007 to 2021 (summarized in the Supplementary file), and none were as inclusive as this review in terms of the period of literature covered, the types of populations included (diversity in parents from different ethnic backgrounds), with no geographical restrictions and as aforementioned, none of the above studies detail parents’ perspective of the influence of cultural values on the uptake of HPV vaccination.

This situation shows that little attention has been given to the effects of culture on the population's judgment-making processes. It can be argued that the consideration of cultural differences is essential when promoting HPV vaccination in cross-cultural populations across the world.^9^ Additionally, it is supported by the WHO’s Immunization Agenda 2030 which was designed to elevate vaccination uptake by tackling every population worldwide to fully benefit from vaccines for good health and well-being.^10^ Therefore, an analysis of studies explicitly reporting on cultural values is required. For this purpose, this review systematically analyzed the qualitative data to examine the potential for cultural discrepancies in multi-cultural parents in the judgment of HPV vaccination for their daughters which may not be achieved from an analysis of quantitative studies.

Individual qualitative studies usually have limited leverage on the development of policies due to their small sample sizes. The particular features of the studied population and the subjective interpretations of the data can lead to concerns about the study's generalisability to broader populations.^11^ The current researchers believe that one way to overcome this perceived limitation is to synthesize information from numerous qualitative studies. This integration of information from various qualitative studies exploring parental perspectives on the influence of cultural values on the uptake of HPV vaccination by their daughters may present a series of themes that can be identified across countries over varying periods.^12^

## Methods

By conducting a qualitative synthesis, the authors add further depth and insight into parents’ views, particularly into the reasoning process involved in utilizing HPV knowledge and information to weigh their daughters’ need for HPV vaccination, under both circumstances, whether the HPV vaccine was included in their national immunization program or a private network. For reporting and synthesizing findings, the guidance outlined by the statement of Preferred Reporting Items for Systematic Reviews and Meta-analyses (PRISMA)^13^ was incorporated throughout the review. The guidelines for enhancing transparency in the reporting of qualitative research (ENTREQ) were then incorporated into the reporting of this systematic review (Supplementary File).^14^

## Search methods

A comprehensive search of electronic databases including CINAHL, PsycINFO, EMBASE, PubMed and Science Direct was conducted in January 2023 to identify eligible studies published in English. The search strategy implemented for the different MeSH terms (depending on the databases), keywords such as “parents”, “daughters”, “papillomavirus vaccines”, “culture” and “qualitative research” were used, and their combinations following Boolean operators OR/AND, wildcard “?”, truncation “*”, and filtering processes to generate the highest quality relevant studies. Hand searches of additional articles were also considered, through identification in reference lists of the included studies based on the inclusion/exclusion criteria. Articles for inclusion included primary studies that reported qualitative analysis of textual data and described the cultural values that influenced intention, experiences, thoughts, views, beliefs, perceptions, feelings, opinions, barriers, and facilitators, which related to psychological, emotional, social, spiritual, religious, or ethnic factors demonstrated by parents towards HPV vaccination for their daughters. The daughter's age was limited to the age eligible for HPV vaccination, especially under the age of 18 where parental consent is prominent in those cases. HPV vaccination offered to female children either free of charge or payable depending on the country's policy was included in the review. Guardians or caregivers of daughters; and male children or sons; young women eligible for HPV vaccination (19 – 26 years old) or very young daughters not eligible for HPV (babies, infants) were excluded from the review. Also, articles excluded were those that reported other childhood vaccinations prescribed to the children; did not report cultural values that influenced intention, experiences, thoughts, views, beliefs, perceptions, feelings, opinions, barriers, and facilitators to HPV vaccination; and non-primary studies (literature reviews, editorials, guidelines, policies, reports, commentaries, letters, minute meetings), mixed methods and purely quantitative studies. Additional information on search strategies is provided in Supplementary Material.

## Quality appraisal and data extraction

Each primary study was appraised using previously validated checklists for qualitative studies, the Critical Appraisal Skills Program (CASP).^15^ The ten items in the appraisal checklist allowed for rapid and accurate evaluation as they are suitable for different types of qualitative reviews.^15^ Studies could receive a maximum of ten points, with higher scores indicating better quality. To ensure rigor, all reviewers agreed that out of ten points, eight points and above, will be considered high-quality studies, seven points as moderate quality studies, whilst those with six points and below were rated low-quality studies.

Currently, there is no universal agreement regarding the quality rating of qualitative studies and subsequent exclusion from the reviews. For this review, studies were not automatically excluded based on overall ‘low quality’ if they contributed relevant qualitative information. However, studies were excluded if the methodology and results were presented in such a way that the findings were insufficient and unreliable to answer the review question despite their achieved quality rating.^16^

The first reviewer (NSS) conducted the assessment, and it was then confirmed by the second reviewer (CHY). Both reviewers assessed each study independently and then met to come to a consensus for any score discrepancies. Table 1 details the results of the methodological appraisal of the studies.

**Table 1:** Methodological quality rating of selected studies using the critical appraisal skills program (CASP).

	Quality criteria											
	1	2	3	4	5	6	7	8	9	10	Total	Quality
Questions^a^	Was there a clear statement of the aims of the research?	Is a qualitative methodology appropriate?	Was the research design appropriate to address the aims of the research?	Was the recruitment strategy appropriate to the aims of the research?	Was the data collected in a way that addressed the research issue?	Has the relationship between researcher and participants been adequately considered?	Have ethical issues been taken into consideration?	Was the data analysis sufficiently rigorous?	Is there a clear statement of findings?	How valuable is the research?		
Bair et al. 2008	1	1	0.5	1	1	1	1	1	1	1	9.5	High
Chan et al. 2023	1	1	1	0.5	1	1	1	1	1	1	9.5	High
Dailey 2013	1	1	0	1	1	0.5	0.5	1	1	1	8	High
Fernandez et al. 2014	1	1	0	1	1	0.5	1	1	1	1	8.5	High
Forster et al. 2017	1	1	0	1	1	0	1	1	1	1	8	High
Galbraith-Gyan et al. 2017	1	1	1	1	1	1	0.5	1	1	0.5	9	High
Gordon et al. 2011	1	1	1	1	1	1	1	1	1	0.5	9.5	High
Gottvall et al., 2013	1	1	1	1	1	0.5	1	1	1	0.5	9	High
Grandahl et al. 2014	1	1	1	1	0.5	0.5	1	1	1	0.5	8.5	High
Madhivanan et al. 2009	1	1	0	1	1	0.5	1	1	1	1	8.5	High
Marlow et al. 2009	1	1	1	1	1	0.5	1	1	1	1	9.5	High
Morales- Campos et al. 2013	1	1	0	1	1	0.5	1	1	1	1	8.5	High
Mupandawana & Cross 2016	1	1	1	1	0.5	0.5	1	0.5	1	0.5	8	High
Netfa et al. 2021	1	1	0	1	1	1	0	1	1	1	8	High
Niccolai et al. 2014	1	1	1	1	1	1	1	1	1	0.5	9.5	High
Salad et al. 2015	1	1	1	1	1	0.5	1	1	1	1	9.5	High
Siu 2014	1	1	1	1	1	0.5	1	1	1	1	9.5	High
Stephens & Thomas 2012	1	1	0	1	1	0	0.5	1	1	1	7.5	Medium
Thompson et al. 2012	1	1	1	1	1	0.5	1	1	1	1	9.5	High
Waller et al. 2006	1	1	1	1	1	0.5	1	0.5	0.5	0.5	8	High
Wong 2009	1	1	1	0.5	1	0.5	1	1	1	1	9	High
Zach & Bentwich 2022	1	1	1	1	0.5	1	1	0.5	1	1	9	High

Keynotes: 1- Yes, 0.5- Can't tell/partially addressed, 0- No.

a Source taken from: CASP (2013). *CASP Checklists*. Critical Appraisal Skills Program, Oxford. Available from http://www.casp-uk.net/casp-tools-checklists on 10th Nov 2022.

The data extracted was based on an extraction form agreed upon by both reviewers. The parameters contained in the form included demographic information, participants’, study characteristics, and key findings (Table 2). Data extraction was independently done by NSS and CHY.

**Table 2:** Summary characteristics of included studies (*n* = 22).

Author/Year/Setting	Aim	Participants	Key findings reported by authors	Key themes identified by reviewers
Bair et al. (2008), United States	To describe Latina mothers’ acceptance of the HPV vaccine for their daughters and explore their knowledge base regarding HPV- related issues	40 mothers	*“I****do not know much****about this disease, because sometimes****we as Hispanics****. Sometimes the resources do not come easy so we can learn and know more about the diseases.”; the* desire to provide “security” for their daughters and the concept of **their daughters belonging to the “*risky youth of this generation***.”; mothers did express concern about the implicit **encouragement of sexual activity.**	External influences Obligation Sexuality-related
Chan et al. (2023), China	To explore the barriers and facilitators influencing South Asian minority and Chinese mothers’ decisions to vaccinate their daughters against HPV in Hong Kong	85 mothers	Believed that their **daughters would remain sexually inactive** before marriage; *“It is ok for the girls to [****wait to] get this vaccination until 18 years old****if they are not married…I mean, if they don't have any sexual life.”*; Most South Asian mothers encountered **language barriers** that limited their ability to access healthcare services.	Upbringing Sexuality-related External influences
Dailey (2013), United States	To understand how parents make the decision to vaccinate or decline vaccination of their children against HPV	16 mothers	Several participants described the vaccine as the **vaccine “for the bad girls”;** vaccine uptake could be interpreted by their children as **parental approval of sexual activity outside of marriage and/or of promiscuity;***If it's safe—in my idea****I want to focus on the good health****.”;* participants who intended on vaccinating her son in the future, discussion of the vaccine would emphasize the long-term health benefits and draw upon **Islam's stance on health**, *“I'm going to tell her****this is for health. This is part of Islam****. The Quran is teaching us to go after good health”*	Vaccine-related Sexuality-related Obligation
Fernandez et al. (2014), Puerto Rico	To explore factors associated with HPV vaccination uptake decisions among Puerto Rican mothers and daughters	9 mothers	Mothers with unvaccinated daughters were more likely to discuss reasons why their daughters were not at risk, including **their belief that they were not sexually active,** “*our family****values are very different****from those of the girls I teach”; “I have doubts, I****talk with my cousin and she says that side effects****are still unknown, that it is not safe, and that we have to wait.”;* Some mothers worried that HPV vaccination would **send daughters the message that it was “ok” to have sex.**	Upbringing External influences Sexuality-related
Forster et al. (2017), United Kingdom	To explore the factors that prevented ethnic minority parents from vaccinating, compared to white British nonvaccinating parents and vaccinating ethnic minority parents	33 participants (1 father; 32 mothers)	Parents from Somali, Bangladeshi, mixed Asian, and white British backgrounds **perceived their daughters to be at low risk** as they **would not be promiscuous or have unprotected sex;** nonvaccinating ethnic minority and White British parents felt concerned that HPV vaccination would **encourage unsafe sexual practices;** ethnic minority parent mentioned a **preference to not use medicines** in general, one of whom had never given their child vaccines.	Upbringing Sexuality-related Vaccine-related
Galbraith-Gyan et al. (2017), United States	To explore the influence of culture on African American mothers’ and daughters’ HPV vaccine acceptance using the PEN-3, a culturally centered conceptual framework	28 mothers	*“The only way my religious beliefs affect me would be that I know the Bible says you****should not have sex until you are married.****Do I think that's realistic in this day and age? Hardly …”*; These mothers reported the belief in **religious doctrine against premarital sex**, and therefore believed HPV vaccination was unnecessary.; *“****I speak to my girlfriend, and she recommended****that I get it for her [her granddaughter]. Ninety-nine percent of the time, I take her advice. She's a nurse, so in her field of work she recommended it and for her children as well.”*	Obligation Upbringing External influences
Gordon et al. (2011), United Kingdom	To explore attitudes to HPV vaccination and reasons for accepting and declining vaccine in the British Jewish community	20 mothers	One mother became **interested in alternative medicine** while her children were still young and did not complete the full vaccination schedule for them; mothers described being wary of vaccines due to previous **negative experiences in their family or social circle;***“It's a lot****less likely in the Jewish community****if your husband or partner has not slept around and therefore the chances of being, of getting it… is much reduced risk and I've therefore not taken it as a serious issue for me, and****hopefully for my kids****”*; Some mothers were concerned about **discussing sexual matters** with their daughters at this age.	Vaccine-related External influences Upbringing Sexuality-related
Gottvall et al. (2013), Sweden	To explore how parents reason when they accept HPV vaccination for their young daughter and also their views on HPV-related information	27 participants (4 fathers; 23 mothers)	*“I have myself had cervical cancer, so I think there is even more reason that my daughter will be vaccinated.****There was no doubt, just a YES****.”; “I think that it might be****too early****in middle school, but definitely in secondary school…seventh grade.”*; They also felt **it was difficult to talk about it and did not know when was a good time** to talk about the sexual transferability of the virus and protection against STIs; Many parents wanted to talk to their daughters about **other preventive methods** for cervical cancer, such as condom use and pap-smear exam.	Obligation Sexuality-related Vaccine-related
Grandahl et al. (2014), Sweden	To explore why parents refused to allow their 10- to 12-year-old daughters to receive HPV vaccination from the Swedish school-based vaccination program	25 participants (2 fathers; 23 mothers)	The parents believed that it would be **several years before she would become sexually active; Recommendations from significant others**, such as family, friends, or healthcare professionals, had an impact on some parents’ decision not to vaccinate; The vaccine was not needed because the daughter was **only supposed to have one partner** and was **not going to lead that kind of life of lax morals.**	Sexuality-related External influences Upbringing
Madhivanan et al. (2009), India	To investigate attitudes toward HPV vaccination among parents of adolescent girls in Mysore, India	44 participants (21 fathers; 23 mothers)	*“We know it is****our duty****to take our children to the hospital to get vaccination.”;* While many parents found HPV vaccination to be acceptable, most **did not support vaccinating young girls between the ages of 9 and 15 years;** many parents felt their own daughters were **unlikely to have sex prior to marriage**; Some urban Hindu mothers suggested that it was necessary to **discuss the vaccine within the family.**	Obligation Sexuality-related Upbringing External influences
Marlow et al. (2009), United Kingdom	To explore attitudes to HPV vaccination among black and Asian mothers living in Britain	20 mothers	Mothers from African backgrounds felt their attitudes to vaccination were **related to their ethnic background** because it meant they ‘have more experiences of the bad side’ and do not take vaccinations for granted; Several of the black Caribbean mothers felt that agreeing to HPV vaccination at 12/13 would be **‘giving a message of promiscuity’** to their daughters, Some also said **they asked people** they knew **with medical backgrounds** whether they would ‘recommend’ it.	Racial/ethnic disparities Sexuality-related External influences
Morales-Campos et al. (2013), United States	To assess Hispanic mothers’ and girls’ perceptions about cervical cancer, HPV, and HPV vaccine	24 mothers	Mothers and girls discussed whether receiving the vaccination implies that **parents condone their daughters’ becoming sexually active;** If the vaccine meant **protecting their daughters’ health and life, mothers indicated they would find a way** to pay for it; Mothers believed Hispanic parents’ **lack of self-confidence, trust, and communication** with their daughters may put them at risk for HPV and cervical cancer.	Sexuality-related Obligation External influences
Mupandawana & Cross (2016), United Kingdom	To explore factors influencing UK-based African parents’ acceptance or decline of the HPV vaccine	10 participants (5 fathers; 5 mothers)	Parents were generally **uncomfortable about discussing a sexual health issue** with their daughters; *If it was up to me, I would consent, but****I am not the head of the family****. Her father said no”;* some refusing the vaccine and deciding to **focus more on talking to their children** about the dangers posed by risky sexual behavior; The fact that the mothers they would normally **discuss vaccination with would be from similar backgrounds,** and thus share similar views.	Sexuality-related Obligation Vaccine-related External influences
Netfa et al. (2021), Australia	To explore the knowledge and attitudes of parents from Arabic backgrounds toward HPV vaccination offered to their children in the national school-based vaccination program	15 mothers	*“I****can't understand it 100 % sure****, if I read it in Arabic, it would be cleared for me.”; “I saw Halal at the top of the information sheet, the****vaccine components are Halal****.”; “I would not give it to my daughter though as son is different to daughter, I cannot guarantee that my son will not do anything because he has the freedom to come and go, a****daughter stays under the auspices of her parents****, she does not come and go alone, and she knows that this thing is not part of our religion.”*	External influences Vaccine-related Upbringing
Niccolai et al. (2014), United States	To explore parents’ attitudes and beliefs about STI and cancer prevention in the context of HPV vaccination using qualitative research methods	34 participants (7 fathers; 27 mothers)	*“I****would love that she be protected****so she wouldn't get it [an STI], you know. We talked about that to her, you know, and her mind open a little bit.”*; Parents also realized that **adolescent sexual activity was sometimes unexpected** and that they may not know when their children become sexually active.; In some instances, parents recognized that their **children may prefer to talk to a doctor rather than a parent**.	Obligation Upbringing Sexuality-related
Salad et al. (2015), Netherland	To explore the perceptions of Somali women living in the Netherlands regarding measures to prevent cervical cancer	32 mothers	*“We do not understand the situation of this country. We****do not understand what is written in Dutch****in the letter”*; Mothers **tend to accept the views of their Somali peers;** Susceptibility to HPV is perceived to be low for Somali girls because they are expected to **not engage in premarital sex**; However, there is also an individual **religious responsibility to improve one's health and prevent disease.**	External influences Upbringing Obligation
Siu (2014), China	To investigate the perceptions of Hong Kong mothers about vaccinating their daughters against HPV	35 mothers	Another common perception among the sampled mothers was that the vaccination had the potential to **encourage premarital sexual behavior;***“Cervical cancer is caused by sex, so I do not think that receiving the vaccination can help you to prevent cervical cancer. I think that****not having sex should be the best way to prevent cervical cancer rather than receiving the vaccination****”; “Only those who are promiscuous need to be vaccinated. I****trust that my daughters will be morally behaved and be good women****when they grow up”*.	Sexuality-related Vaccine-related Upbringing
Stephens & Thomas (2012), United States	To identify immigrated Haitian mothers’ beliefs about HPV vaccination and cultural factors that influence their willingness and resistance to having their daughters vaccinated	31 mothers	One noted its uselessness because she “knew” her daughter **would wait to have sex until she was married;***“I think young girls would****think they can [have sex]****and****not worry if they get [the HPV vaccine]****“;* Six mothers were concerned that the vaccination may be used as a reason **to discriminate against their daughters;** They recalled the **stereotyping of Haitians as disease carriers** at the peak of the HIV/AIDS crisis, which leads to an increase in immigration-related **barriers and social stigmas.**	Upbringing Sexuality-related External influences
Thompson et al. (2012), United States	To describe the cultural attitudes, social and environmental factors that affect African American parents’ intent to vaccinate their daughters against HPV	30 participants (5 fathers; 25 mothers)	Parents noted that **they would see more urgency** to HPV vaccination if they believed that **their daughters were sexually active;***“I guide them, even my son. You know, I talk about****abstinence all the time****. So, guide them”;* Parents expressed reluctance to vaccinate girls as **young** as nine years old. Parents stated that they were **less concerned about sexuality at this age**; “*I'm led by the Holy Spirit.****It led me to make the right decision that this would help her, you know, then yeah****”*	Upbringing Sexuality-related Obligation
Waller et al. (2006), United Kingdom	To explore mothers’ attitudes towards vaccination	24 mothers	Women in group 3 seemed **more in favor of other forms of cancer prevention**, such as screening and lifestyle change; attitudes were generally favorable, with women acknowledging that their children **would one day be sexually active and wanting to protect** them if possible; They expressed the view that vaccinating children against STIs would be ‘‘teaching them …that **it's okay to be promiscuous’’;** Another also thought that her daughters were **less at risk** than others because they **did not *‘‘hang around****on the streets like I see some kids do”*	Vaccine-related Obligation Sexuality-related Upbringing
Wong (2009), Malaysia	To assess mother's knowledge and attitude toward HPV vaccination	47 mothers	Most of them **wanted to do the best** that they could to protect their daughter, *“Prevention is better than cure, as a mother as much as possible****we want the best for our daughter****”;* Many viewed that their children were **not likely to be sexually active at the recommended age** and therefore will not immediately benefit from the vaccination at present moment; The less educated Muslim mothers would prefer to hold immunization as they **believed that they have imparted proper moral and religious education** to their children about the consequence of premarital sex; Mandatory vaccination would alleviate the **promiscuity stigma** associated with receiving the vaccine; Most of the Muslim respondents were concerned that because it is a western product and related to an STD, the vaccine **may contain alcohol or may be made from nonhalal sources.**	Obligation Sexuality-related Upbringing External influences Vaccine-related
Zach & Bentwich (2022), Jerusalem	To better understand the basis for members of an ultra‐Orthodox Jewish community to object to the HPV vaccine and how such objections can and cannot be reduced, thereby improving cultural competence—namely, the cultural understanding and ethical addressing of HPV vaccination refusal	10 mothers	*“It [the HPV vaccine] is not relevant…****we do not have these risk factors,****thank God.”; “If, for some reason, my daughters will have multiple partners [before marriage], then I will prepare them and will****teach them about birth control****.”; “Originally, I thought not to vaccinate my daughters and not even to talk about it with them, but today I say to myself—it could be that****when they reach the age of 18–19 I will think about encouraging****them to get vaccinated.”*	Upbringing Vaccine-related Obligation

### Data analysis and synthesis

A data extraction framework was adapted from Bettany-Saltikov's work, in which the reviewers highlighted relevant information: author(s), year, aim(s), participants, settings, methodology and key findings.^17^ All papers included were read independently (NSS and CHY), and a cross-checking process was done with the third reviewer (KLA). During this stage, the outcome data were extracted which comprised all the texts under the headings of ‘results’ or ‘findings’, and they were then transferred to a data extraction form.

Each study's finding was synthesized using Thomas and Harden's thematic synthesis method and the stages commenced with line-by-line coding of text findings and organization of coding into descriptive and analytical themes.^18^ Any discrepancies were discussed between the reviewers (NSS, CHY, and KLA) and changes to the themes were made as necessary in consideration of the original findings. Subsequent studies were coded into pre-existing concepts, and new concepts were created when deemed necessary.

### Protocol and registration

The protocol for this systematic review has been registered with PROSPERO (CRD: 42020211324) and can be accessed at.^19^

## Results

**Figure 1:**
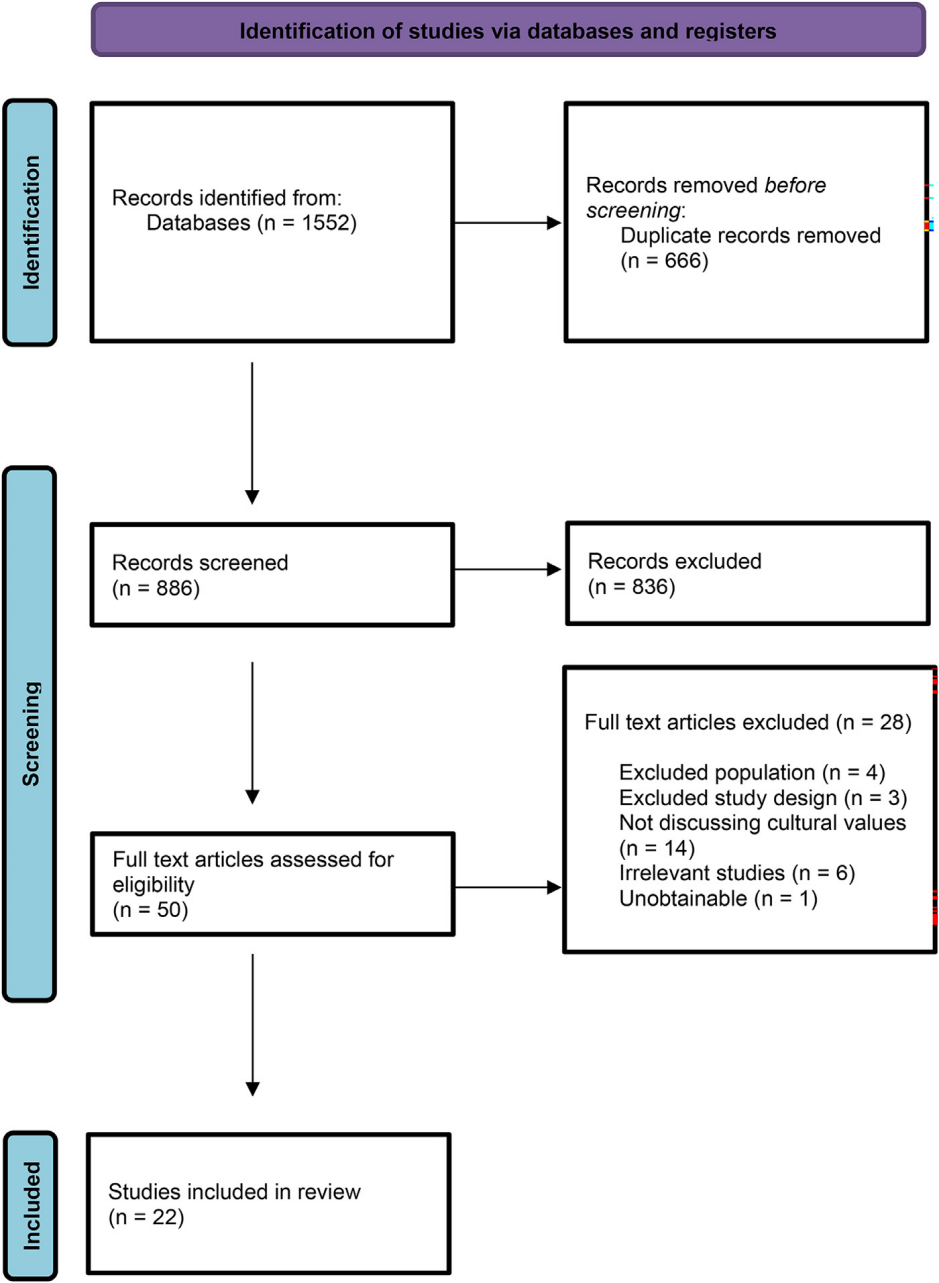
PRISMA flow diagram of studies’ identifications, screening, eligibility, and selection process. *From*: Page, M.J., McKenzie, J.E., Bossuyt, P.M., Boutron, I., Hoffmann, T.C., Mulrow, C.D., … Moher, D. (2021). The PRISMA 2020 statement: An updated guideline for reporting systematic reviews. *BMJ, 372*, n71. doi: 10.1136/bmj.n71.

The PRISMA flow diagram illustrates the search and selection process (Figure 1). The search returned 1552 citations. Following the removal of duplicates, reviewers (NSS and CHY) screened all titles and abstracts for relevancy. A further 836 articles were excluded as they did not meet the inclusion criteria for the review following the title and abstract. When the abstract was not descriptive enough, or no abstract was available, the full text was read, providing a total of 50 full-text papers that were obtained and reviewed. Inclusion criteria were applied to the 50 studies, and 28 studies were excluded, leaving 22 studies eligible for inclusion.

In total, 22 studies were included comprising a total of 639 participants from various ethnic backgrounds. Nineteen studies included specific ethnic minority participants, and three studies included participants from the general population.^20^, ^21^, ^22^ They were Asian Chinese (120 participants), Asian/Asian-British (106 participants), Hispanic and Latino (73 participants), Black/African/Caribbean/Black-British backgrounds (70 participants), Somali (49 participants), Indian (45 participants), White British (39 participants), Haitian (31 participants), Arabic (15 participants), Non-British White (8; included Austrian, Italian, South African, Lithuanian, American, Hungarian, German and mixed British/ Finnish = 8 participants), Bangladeshi (6 participants), and Sri Lanka Tamil (1 participant), and the final 76 participants were drawn from a general sample of a Western population.^20^, ^21^, ^22^ All the included studies aimed to explore the factors influencing parents’ willingness or resistance to their daughters being vaccinated against HPV. Two studies exclusively reported on the factors that prevented parents from vaccinating their daughters.^7^^,^^22^ See Tables 2 and 3.

**Table 3:** Methodology characteristics of included studies (*n* = 22).

Author/Year	Sampling methods	Data collection	Data analysis
Bair et al. (2008)	Convenience	Face-to-face interviews	Thematic content analysis
Chan et al. (2023)	Purposive	Semi-structured focus groups	Thematic content analysis
Dailey (2013)	Purposive	Semi- structured interviews	Grounded theory approach
Fernandez et al. (2014)	Purposive	Brief demographic questionnaire and focus groups discussion	Grounded theory approach
Forster et al. (2017)	Purposive	Interviews	Framework analysis
Galbraith-Gyan et al. (2017)	Convenience	Semi- structured interviews	Grounded theory approach
Gordon et al. (2011)	Purposive	Face-to-face interviews	Framework analysis
Gottvall et al. (2013)	Convenience	Interviews	Thematic content analysis
Grandahl et al. (2014)	Purposive	Brief demographic questionnaires and face-to-face interviews	Latent content analysis
Madhivanan et al. (2009)	Convenience, snowball	Focus groups discussion	Framework analysis
Marlow et al. (2009)	Purposive, snowball	Face-to-face interviews	Framework analysis
Morales-Campos et al. (2013)	Purposive	Brief demographic questionnaires and focus groups discussion	Grounded theory approach
Mupandawana and Cross (2016)	Purposive, snowball	Semi- structured interviews	Thematic content analysis
Netfa et al. (2021)	Snowball	Face-to-face interviews	Thematic content analysis
Niccolai et al. (2014)	Convenience	Semi- structured interviews	Thematic content analysis
Salad et al. (2015)	Purposive, convenience, snowball	Semi- structured interviews, natural group discussion	Thematic content analysis
Siu (2014)	Purposive	Individual semi- structured interviews	Grounded theory approach
Stephens and Thomas (2012)	Convenience	Brief demographic questionnaires and semi- structured interviews	Thematic content analysis
Thompson et al. (2012)	Purposive, community sampling	Self- administered survey and semi- structured interviews	Not reported
Waller et al. (2006)	Purposive, snowball	Focus groups discussion	Framework analysis
Wong (2009)	Purposive	Brief demographic questionnaires and focus groups discussion	Grounded theory approach
Zach and Bentwich (2022)	Purposive	Semi- structured interviews	Thematic content analysis

**Figure 2:**
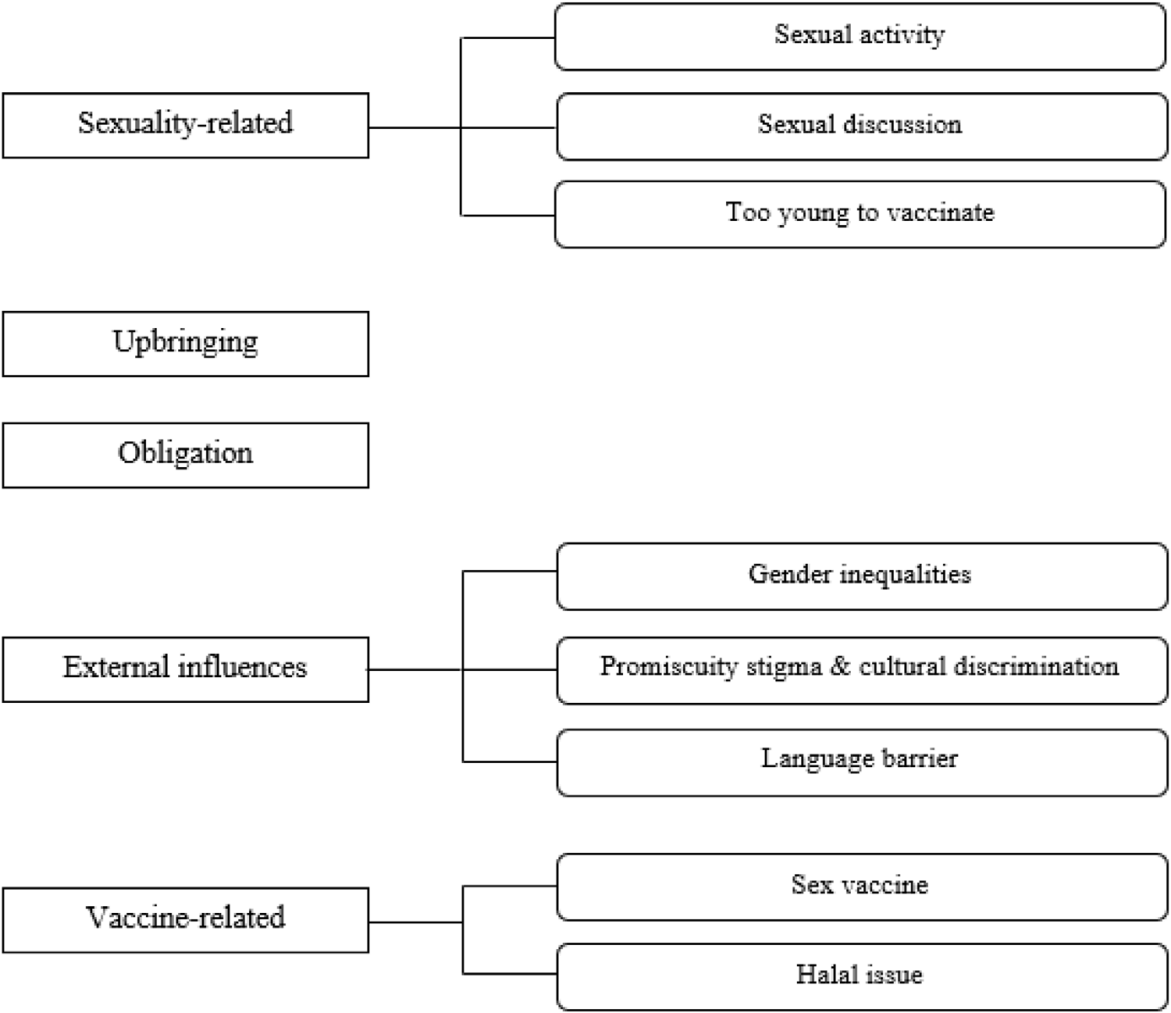
Development of core themes and domains.

Thematic synthesis of each included study resulted in the emergence of five prominent themes related to parents’ perspectives of how the influence of cultural values affected their decision whether to vaccinate their daughters against HPV. These were: 1) sexuality-related concerns; 2) upbringing and moral values; 3) obligation to protect; 4) external influences; and 5) vaccine-related concerns. The development of themes and domains is presented in Figure 2.

## Theme 1: Sexuality-related concerns

Researchers have uncovered evidence demonstrating how culture and society shape sexuality concerns.^23^, ^24^, ^25^ Culture provides a substantial context for comprehending sexuality. The values, beliefs and behaviors linked to an individual's sexuality also offer substantial insight into the broader beliefs and values of the society they are part of or where they come from. In this review, parents expressed their concerns about sexually related issues linked to the uptake of HPV vaccination for their daughters. It includes three subthemes: 1) sexual activity, 2) sexual discussion, and 3) too young to vaccinate.

### Subtheme 1.1: Sexual activity

This subtheme emerged in 12 included studies (55 %),^4^^,^^6^, ^7^^,^^10^^,^^22^^,^^26^, ^27^, ^28^, ^29^, ^30^, ^31^, ^32^ in which parents were concerned about the link between HPV vaccination and sexual activity. However, there were mixed opinions about parental concerns about risky sexual activity among their daughters if they are vaccinated. Of these 12 studies, one study,^30^ indicated that parents did not believe that HPV vaccination would encourage early sexuality of their daughters.

“The shot (is) not a hormone shot; it will not make them want to have sex”^30^

Meanwhile, in the other 11 studies, parents expressed concerns that the HPV vaccine might encourage earlier sexual debuts or promote promiscuity in their children due to the misinterpretation of protection offered by the vaccine.

“Now she will become sexually active because she is vaccinated.”^31^

### Subtheme 1.2: Sexual discussion

Many parents were concerned that discussing HPV with their daughters meant having to address sexual health issues, as sexual transmission was conclusively linked to HPV infection. Eleven studies (50 %) mentioned that parents were avoiding or expressing the intention to avoid discussion of the vaccine and sexual health with their children.^4^, ^5^, ^6^^,^^20^^,^^21^^,^^27^^,^^29^, ^30^^,^^33^, ^34^, ^35^

Of these eleven studies, five studies reported that parents were generally uncomfortable being engaged in a sexuality-related conversation, and therefore would not discuss HPV vaccination because of its link with sex.^5^, ^6^^,^^27^^,^^29^^,^^34^ Furthermore, Marlow et al.^27^ and Galbraith-Gyan et al.,^34^ reported that several mothers explained that sex-related discussion was considered ‘taboo’ in some cultures and that this made discussions about the vaccine and associated sexuality difficult between couples.

However, another six studies mentioned that parents did not prefer to discuss the vaccination at a younger age and did not think their children were mature enough to be involved in the sexual discussion, but they did foresee having discussions with them at a more developmentally appropriate time.^4^^,^^20^, ^21^^,^^30^^,^^33^^,^^35^

“I don't wanna talk about that one. She's too young for that. When they older—yah I will tell her”^4^

### Subtheme 1.3: Too young to vaccinate

Culturally, parents believed their children were too young to start their sexual life, and therefore, they perceived HPV vaccination as being unnecessary. Fifteen studies (68 %) mentioned that parents expressed their concerns about the age of sexual activity and felt that their daughters were too young to be vaccinated against STIs.^5^, ^6^^,^^20^, ^21^, ^22^^,^^27^, ^28^, ^29^, ^30^, ^31^, ^32^, ^33^^,^^36^, ^37^, ^38^ Although they were aware of the need to vaccinate children before they became sexually active, some women were adamant that they would not vaccinate their daughters at a younger age.

“My daughter is still very young, so I think that there is no need for her to be vaccinated at the moment. It is too early for her; she is just 11 years old now!”^32^

However, a subset of parents in four studies^22^^,^^28^^,^^31^^,^^38^ thought that it was preferable to hold off on vaccination until their daughters were older and that it was ideal to vaccinate daughters during their late adolescence or just before they start high school or college.

“And then we feel, well, she is just twelve and not sexually active. She is still just a girl, so we feel that we can vaccinate her later if we feel that there is a need for it”.^22^

## Theme 2: Upbringing and moral values

Parental views of good cultural upbringing with higher moral codes and religious practices are also influential in their decision-making on vaccinating their daughters against HPV. A consensus emerged from 15 studies (68 %), on the perception that; upbringings with higher moral codes or religious beliefs were thought to shape a child's behavior and therefore reduced the risk of acquiring HPV, due to good moral behavior.^6^^,^^9^^,^^22^^,^^26^, ^27^, ^28^^,^^30^, ^31^^,^^33^, ^34^, ^35^, ^36^, ^37^, ^38^, ^39^

Religious practice such as abstaining from premarital sex was likely the main reason why parents with unvaccinated daughters believed HPV infection ‘wouldn't be a worry’. There was a consensus on this issue across several cultures and religions.

“I've also been told that ‘religious’ women are less likely, one of the cancers we're less likely to get, if you sleep with men who've been circumcised, or use a condom or both, and stay with the same partner who is hopefully not fooling around, you've got less chance of getting, not no chance, but it's lowered”^33^

“Coming from a ‘religious’ background… we don't have sex before marriage for example, so your first experiences of these things are when you're married and you stay in a relationship… because of that reason I'd probably say no, I wouldn't bother with it with my two girls”^27^

A mother also expressed her feelings this way:

“Girls do not need until they are older. I will educate them, and they will do what is right. No one in my family has an illegal sexual relationship with anyone. Not my father or my mother. My children know about this, and they will do what is right.”^36^

## Theme 3: Obligation to protect

This theme emerged from the literature as parents mentioned the views on the link between the obligation to protect their daughters due to the culture they lived in, and the acceptance or decline of HPV vaccination for their daughters.

In seven studies, (32 %), a small number of parents felt that the receipt for vaccination against STIs would depend on the culture they lived in.^20^^,^^26^, ^27^^,^^30^^,^^33^, ^34^^,^^40^ The findings indicated that parents noted more urgency to vaccinate if they felt that their daughters lived in a culture where they were sexually active or ‘messing around with local boys’.^20^^,^^27^^,^^33^, ^34^^,^^40^ Therefore, parents opted to vaccinate against HPV if they believed that they could not control their children's behavior. A mother in a study echoed:

“It's very good that it does that [prevents STI] especially for umm, adolescents that, you know, in this day and age where they like to, you know, have sex, you know, young teenagers and getting pregnant and you know…A lot, you know, a lot of diseases that are out there…’Cause this day and age, these young kids think it's OK to have unprotected sex…They all think it's OK but they don't realize the consequences of all the diseases that are out there”^40^

## Theme 4: External influences

This theme emerged from the data as parents echoed the external influences or cultural norms that had affected their decision to consent to HPV vaccination for their daughters. These influences emerged in the form of three subthemes: 1) gender inequalities, 2) promiscuity stigma and cultural discrimination, and 3) language barrier.

### Subtheme 4.1: Gender inequalities

Masculine power dynamics of fathers were found in three studies (14 %)^6^^,^^36^^,^^38^ that contributed to the decision either to consent to or decline the vaccine. It was evident from the parents in those studies that fathers, rather than mothers, were the ultimate decision-makers in most family matters, especially those concerning the children. In certain countries, culture has generally placed women under their husbands’ authority may result in a major barrier to vaccinating their daughters.^6^ This cultural value disempowers mothers and makes it difficult for them to consent to vaccination without approval from their husbands. Some even feared being divorced by their husbands as a result.

“If I consented against his wish and something happened to her, or he found out about it, he would divorce me. Accuse me of being promiscuous and rebellious”^6^

### Subtheme 4.2: Promiscuity stigma and cultural discrimination

Parents who had concerns about promiscuity stigma by society were undecided or less likely to vaccinate in four studies (18 %).^6^^,^^28^, ^29^^,^^38^ Mothers were concerned that the stigma of promiscuity caused by vaccination may lead to discrimination against their daughters. Also, they were worried that as members of a marginalized population, they were vulnerable to unfair research practices if they opted to vaccinate their daughters.

“Of course, in our society, very few people have knowledge about it; they will think in a negative way. So, they'll say, oh this is a sexual disease and transmitted through sex, and so they might have relations with boys and something like that”^23^

“Remember this is a white man's vaccine. The white man brought us AIDS to kill us off because we were too many; now, they might want to make our daughters sterile”^6^

### Subtheme 4.3: Language barrier

In three studies (14 %),^37^, ^38^, ^39^ mothers thought that language issues made it difficult for them to get their children vaccinated. Consequently, the mother's lack of knowledge and lack of language skills largely influence their decision of whether to consent to vaccination.

“There are medical terms that are hard to understand in the English language, but in Arabic language, I can read it and sign on it. Many times, my daughter brings letters from school, it is written in English language, and I don't know what is in it”^39^

## Theme 5: Vaccine-related concerns

A consensus on the vaccine-related concerns that influenced HPV vaccination receipt for eligible daughters has been expressed by minority parents. These concerns are culturally related and emerged as subthemes: 1) sex vaccine, and 2) halal issue.

### Subtheme 5.1: Sex vaccine

Conservative cultural background influenced several participants to view the vaccine as the vaccine “for the bad girls” or “women who are promiscuous or engage in some sort of wild sexual behavior”, thus influencing their decisions to not vaccinate as mentioned in three studies (14 %).^4^^,^^6^^,^^32^ Most parents continued with the misperception that only sexually active and promiscuous girls need to be vaccinated.

“Of course! A vaccine for prostitution? She will become a whore, everyone's horse”^6^

### Subtheme 5.2: Halal issue

Whether the vaccine was halal or permissible for some religions to consume seemed to be an important consideration for some parents in two studies^28^^,^^39^ (9 %). Most were concerned about the contents of the vaccine on cultural and religious grounds, as the vaccine may contain alcohol or made from nonhalal sources and many stressed that they would reject the vaccine if it were nonhalal.

“If nonhalal (kosher), I won't give it to my daughter, halal is important to us. We want to know the ingredient, is it halal? If darurat (in situations of exigency) ok, but this vaccine is for prevention only, not treatment”^28^

## Discussion

The present study aimed to review recent literature on how a daughter's uptake of HPV vaccination is shaped by their parents’ cultural perspectives. Five prominent themes (sexuality-related concerns, upbringing and moral values, obligation to protect, external influences, and vaccine-related concerns) emerged from the data.

### Theme 1: Sexuality-related concerns

Sexuality-related concerns are core aspects of parental decisions merged from this systematic review, and this review suggests that these concerns influence parental HPV vaccine acceptance and inhibition. These concerns included the promotion of sexual activity, the difficulty of sexual discussion, and that the daughters were too young to be vaccinated. The link between HPV vaccination and sexual activity can be considered the most argumentative issue as it caused a great deal of debate among parents in most of the included studies. This is consistent with previous research indicating that some parental resistance to HPV vaccination was rooted in the belief that it was linked to their daughter's sexual activity thus affecting vaccine uptake.^18^^,^^41^ Given that HPV's primary mode of transmission is through intimate skin-to-skin or sexual contact,^42^ parents often perceive potential risks by encouraging vaccination. They indicated that this may be seen as sanctioning their daughters’ early sexual debut, risky sexual behaviors, and promoting promiscuity in their children. These beliefs of promoting sexual disinhibition are consistent with previous studies.^41^^,^^43^, ^44^ However, HPV vaccination has been proven inaccurate in promoting sexual activity as noted in previous literature.^45^, ^46^ Parents expressed these concerns due to the misinterpretation of the protection offered by vaccines, and because they felt that vaccinating their daughters could deliver the message that they were implicitly condoning their daughters’ sexual lives at an early age. Numerous studies reported similar sexual complacency concerns as the crucial aspect of vaccine hesitancy among parents.^28^^,^^47^, ^48^, ^49^, ^50^ This concern was particularly prevalent among parents of teenage daughters and therefore suggests that young daughters are greatly influenced and regulated by their parents’ sexual perceptions, as other studies noted.^51^, ^52^

Moreover, some parents were found to avoid or expressed an intention to avoid discussion of the vaccine and sexual health with their children. The findings are consistent with those of other studies.^53^, ^54^ They tended to avoid the discussion before their daughters’ marriage. This avoidance must be squared with the findings that exist among parents with strong religious and cultural beliefs, particularly about abstinence from premarital sex.^27^ Furthermore, a study has shown that ethnic minorities are less likely to talk with others about the vaccine.^55^ The findings of the present systematic review, however, showed divided views with some included studies reporting that parents openly discuss the HPV vaccine with their daughters.^4^^,^^30^^,^^33^ Other previous studies reported similar concerns of openness that exist particularly among Western populations.^56^, ^57^ For instance, research by McRee et al.^56^ showed that US mothers were discussing HPV and sexuality with their daughters and a study by Spencer et al.^58^ noted that parents in Western populations reported making a joint decision with their daughters about HPV vaccination after a discussion with them.

Furthermore, there were concerns about vaccinating young daughters as they were perceived to be too young to start their sexual lives, and thus less likely to be sexually active. This risk perception correlated with young age and daughters’ abstinence and therefore parents were not in favor of vaccination.^46^^,^^59^ They perceived no necessity to consent to vaccination and expressed the intention to hold off on vaccination for their daughters. Yet, as in many other studies,^60^, ^61^ parents did consider vaccinating their daughters at a much later age.

**Theme 2: Upbringing and moral values**

Another key finding of this systematic review is that upbringing with higher moral codes, particularly abstinence from premarital sex, resulted in a perceived low susceptibility to HPV infection, and was seen as a barrier to vaccination as reported in previous studies.^59^^,^^62^ It is important to note that, there was, however, a subset of parents across several cultures and religions who showed a relatively strong antipathy toward the HPV vaccine as a STI vaccine on religious and moral grounds. However, although a subset of parents described religion as influential in their daily lives, it was not how they exclusively made decisions. These findings are similar to other previous studies demonstrating that there is no strong link between religious beliefs and decision-making.^9^^,^^63^ A small number of parents believed that the decision not to vaccinate was right if their daughters were not going to lead a life of lax morals and lived up to their expectations.^22^

Based on the discussion of the review's findings and reference to the existing literature as above, it provides an understanding of the way culture and religion may influence HPV vaccination decisions among parents. It is hoped that the implementation of the vaccine promotion strategies should include the monitoring of ethnicity and cultural values for the success of HPV vaccine promotion among young women, in the future. Therefore, to achieve this, communication between the pediatricians and parents should be enhanced in a way that the pediatricians should focus on building a strong rapport with the parents and employ persistent, forceful language with minimal acquiescence, shifting the conversation focus from sexual activity to cervical cancer caused by HPV infection during health promotion.^64^ Additionally, pediatricians should collaborate with the community to culturally adapt the vaccination language, therefore addressing barriers noted by parents.^64^ These strategies may reduce missed opportunities for HPV prevention and potentially decrease racial and ethnic disparities in HPV vaccination.

## Implications for future research and recommendations

The search returned most of the studies conducted in developed countries, rather than other developing countries or more conservative societies, hence restricting the generalizability to the other part of the world. Further primary research is required in the context of the vaccination program and should include groups that are currently under-represented, such as low socio-economic and ethnic minorities, particularly in low- or middle-income countries (LMICs), to understand parental acceptability of HPV vaccination where the cervical cancer incidences are higher, and HPV vaccine uptake is lacking. As this systematic review clearly illustrates the need to target interventions relevant to cultural concerns to increase vaccination receipt and uptake, this will further identify more areas for future research.

In addition, theoretical frameworks were found to be underutilized in the included primary research. Of the 22 studies included in the review, only three studies^34^^,^^37^, ^38^ reported using theoretical models, which suggests that researchers in this field should be encouraged to utilize theoretical models when exploring the perception of the parents toward HPV vaccination so that the interventions to improve uptake will be more effective.^28^ Finally, given that most existing data was collected from mothers (*n* = 594) compared to fathers (*n* = 45), future studies that focus on HPV vaccine acceptance among fathers should be considered, as fathers are also reported to play a vital role in consenting to vaccination for their daughters, in this review. Considering the importance of fathers in this instance, they should not be underappreciated in any future studies in this area.

## Limitations

Some limitations of this review are acknowledged. The first limitation is the lack of inclusion of quantitative studies in the systematic review where casual inferences could be drawn about the impact of cultural beliefs and attitudes on HPV vaccination among girls. Causal inferences cannot be drawn from qualitative studies. Reporting bias may have taken place since only findings pertinent to the review aims are presented.^65^ Hence, it was attempted to minimize the risk of this error by having a second reviewer cross-check the extracted data.

Moreover, the search returned most of the studies from developed countries, rather than developing countries and more conservative societies, hence limiting the applicability of the findings within these settings. The included studies were also heterogeneous in terms of geographical settings, religion, culture, health system, and period (before and after the FDA HPV vaccine approval). One further possible limitation is the fact that most of the included studies were from the US and UK, which may limit the extent the authors can generalize the findings to other healthcare settings. As such, the potential bias is prominent, and results should be interpreted with a degree of caution in mind. Additionally, the lack of inclusion of studies on adolescent boys is another limitation and limits the generalizability of findings.

## Conclusion

This systematic review has offered some insight into the evidence that cultural values should be considered when seeking to understand the mechanism underpinning parental decision-making, especially about HPV vaccination. The differences in cultural views found affirm the need to monitor ethnicity and cultural values in vaccine promotion. In addition, meticulous consideration of parents’ views is needed as part of the process to ensure vaccine uptake and prevent vaccine hesitancy. Eventually, if vaccination programs or information are designed to target the ethnic-specific concerns and cultural values in the community, alongside those that are addressed by parents in general, vaccination uptake may improve, and the global vaccination gap may be filled.

## Implications for key findings

Pediatricians are professionally bound to promote and empower health outcomes, particularly among young, adolescent women by improving the uptake of vaccination to combat both children's morbidity and mortality caused by HPV infection, including HPV vaccination. The findings of the systematic review have an impact, especially in pediatric community settings. The controversy over the lack of consensus regarding the importance of HPV vaccination among daughters might instill a sense of ambiguity amongst parents. In addition, the findings point to the need to address cultural, religious, and language barriers to improve the acceptability and uptake of HPV vaccination, especially among ethnic minorities. Therefore, a middle ground is hoped to be reached by informing pediatricians who are involved in health promotion to encourage and support parents to vaccinate their daughters and protect them from HPV-related diseases. When pediatricians have a greater understanding, they may offer enhanced information and education to parents. As a result, parents may be influenced and more fully supported by health professionals to make positive choices. To improve the communication between pediatricians and the parents, the pediatrician should build a good rapport between the parents and use persistent, forceful language with minimal acquiescence, shifting the conversation to focus from sexual activity to cervical cancer due to HPV infection during the health promotion (64). Also, pediatricians should collaborate with the community to culturally adapt the vaccination language to overcome the barriers noted by the parents. These methods may reduce the missed opportunities for HPV prevention and potentially decrease racial and ethnic disparities in HPV vaccination.

## Funding

This research did not receive any specific grant from funding agencies in the public, commercial, or not-for-profit sectors.

## Conflicts of interest

None.

## Appendix Supplementary materials

Image, application 1

## Footnotes

Supplementary material associated with this article can be found in the online version at doi:10.1016/j.jped.2024.07.012.
