# Supplementary material for: Cultural barriers and facilitators of the parents for human papillomavirus (HPV) vaccination uptake by their daughters: A systematic review
Source: J Pediatr (Rio J). 2024 Nov 4;101(2):133–49. doi: 10.1016/j.jped.2024.07.012 (PMC11889688; doi:10.1016/j.jped.2024.07.012)
Supplement: Supplementary file 1 [file mmc1.docx]

**JPED-D-24-00027_Supplementary Materials**

**Systematic reviews related to HPV vaccination**

| **Author/ Year** | **Titles** | **Review dates** | **Study types** | **Population types** |
| --- | --- | --- | --- | --- |
| Allen et al. 2010a | A systematic review of measures used in studies of human papillomavirus (HPV) vaccine acceptability | Jan 1995 – May 2008 | Quantitative | Adolescents, young adults and parents |
| Brewer & Fazekas 2007 | Predictors of HPV vaccine acceptability: a theory-informed, systematic review | 1995 – 2007 | Quantitative and qualitative | Adolescents, young adults and parents in the US |
| Chan 2012 | A systematic review of literature about women’s knowledge and attitudes towards human papillomavirus (HPV) vaccination | 2005 – 2009 | Quantitative and qualitative | Females (no age limit) |
| Coles et al. 2015 | The association of human papillomavirus vaccination with sexual behaviors and human papillomavirus knowledge: a systematic review | To 2015 | Quantitative | Any age and gender in the UK |
| Ferrer et al. 2014 | Barriers and facilitators to HPV vaccination of young women in high-income countries: a qualitative systematic review and evidence synthesis | Inception – March 2012 | Qualitative | Policy makers, professionals involved in program, parents, and young women |
| Fisher et al. 2013 | Inequalities in the uptake of human papillomavirus vaccination: a systematic review and meta-analysis | Inception – March 2012 | Quantitative | Primary caregiver of adolescent young women |
| Fu et al. 2014 | Educational interventions to increase HPV vaccination acceptance: a systematic review | 1946 – August 2013 | Quantitative | Adolescents, young adults, parents |
| Garcini et al. 2012 | The study of human papillomavirus (HPV) vaccine uptake from a parental perspective: a systematic review of observational studies in the United States | 2006 – 2012 | Quantitative | US parents of children (no age limit) |
| Galbraith et al. 2016 | Parental acceptance and uptake of the HPV vaccine among African-America and Latinos in the United States | No time restricted | Quantitative and qualitative | Focused on African-Americans and/or Latinos parents or caregivers of adolescents girls aged 9-17 years old in the US |
| Gilkey & McRee 2016 | Provider communication about HPV vaccination: a systematic review | Aug 2015 | Quantitative and qualitative | US sample related to provider communication (patients, parents, and providers) |
| Hendry et al. 2013 | "HPV? Never heard of it!": a systematic review of girls' and parents' information needs, views and preferences about human papillomavirus vaccination | 1980 – Aug 2011 | Quantitative and qualitative | Parents of children aged 9-15, young women up to 26, healthcare professionals |
| Holman et al. 2014 | Barriers to human papillomavirus vaccination among US adolescents: a systematic review of the literature | 2009 – 2012 | Quantitative and qualitative | Healthcare professionals, parents, underserved and disadvantaged populations, and males in the US |
| Kabakama et al. 2016 | Social mobilization, consent and acceptability: a review of human papillomavirus vaccination procedures in low and middle-income countries | Jan 2007 – Jan 2015 | Qualitative | Ministry of Health representatives (EPI team members or equivalent), representatives from partner NGOs, and doctors/researchers |
| Kasting et al. 2016 | Tempest in a teapot: a systematic review of HPV vaccination and risk compensation research | Jan 2008 – June 2015 | Quantitative | Both males and females |
| Kessels et al. 2012 | Factors associated with HPV vaccine uptake in teenage girls: a systematic review | 2006 – 2011 | Quantitative and qualitative | Girls aged 9-18 years |
| Klug et al. 2008 | Knowledge about infection with human papillomavirus: a systematic review | 1982 – 2006 | Quantitative | General public, students, patients and health professionals |
| Madhivanan et al. 2016 | Human papillomavirus vaccination and sexual disinhibition in females: a systematic review | 2004 – 2015 | Quantitative | Girls aged 11 years to women aged 40 years |
| Marlow 2011 | HPV vaccination among ethnic minorities in the UK: knowledge, acceptability and attitudes | 2000 – 2010 | Quantitative and qualitative | Any UK lay population that included an ethnic minority group |
| Mishra 2011 | Implementing HPV vaccines: public knowledge, attitudes and the need for education | 2000 – 2010 | Qualitative | Any age and gender |
| Newman et al. 2013 | HPV acceptability among men: a systematic review and meta- analysis | To March 2013 | Quantitative | Men (no age limit) |
| Newman et al. 2018 | Parent’s uptake if human papillomavirus vaccines for their children: a systematic review and meta-analysis of observational studies | Inception to November 2017 | Quantitative | Children ≤ 18 years |
| Niccolai & Hansen 2015 | Practice- and community-based interventions to increase human papillomavirus vaccine coverage: a systematic review | To July 2014 | Quantitative | Any gender of adolescents |
| Perlman et al. 2014 | Knowledge and awareness of HPV vaccine and acceptability to vaccinate in sub-Saharan Africa: a systematic review | 1990 – 2011 | Quantitative and qualitative | Adolescents, parents/guardians and healthcare workers in sub-Saharan country |
| Radisic et al. 2017 | Factors associated with parents' attitudes to the HPV vaccination of their adolescent sons: a systematic review | Oct 2006 – July 2015 | Quantitative and qualitative | Parents of adolescent sons |
| Santhanes et al. 2018 | Factors influencing intention to obtain the HPV vaccine in South East Asian and Western Pacific regions: a systematic and meta-analysis | Inception – December 2016 | Quantitative | Women eligible for HPV immunization; i.e., girls aged 9–13 years old and girls aged ≥ 15 years old. Parents who had daughter’s eligible for HPV immunization |
| Santhanes et al. 2018 | Factors involved in human papillomavirus (HPV) vaccine hesitancy among women in the South-East Asian Region (SEAR) and Western Pacific Region (WPR): A scoping review | Inception – December 2016 | Quantitative and qualitative | Young adolescent girls between nine to 14 years of age, women aged ≥ 15 years old, and HIV infected or immunocompromised women.  Third party individuals who may influence a woman’s decision in taking the vaccine, such as healthcare workers and female parents |
| Trim et al. 2011 | Parental knowledge, attitudes, and behaviors towards human papillomavirus vaccination for their children: a systematic review from 2001 to 2011 | 2001 – 2011 | Quantitative | Parents |
| Young 2010 | HPV vaccine acceptance among women in the Asian Pacific: a systematic review of the literature | 1995 – 2010 | Quantitative and qualitative | Women in the Asian Pacific region |

**PRISMA 2020 checklist**

| **Section and Topic** | **Item #** | **Checklist item** | **Location where item is reported** |
| --- | --- | --- | --- |
| **TITLE** | | |  |
| Title | 1 | Identify the report as a systematic review. | Page 1 |
| **ABSTRACT** | | |  |
| Abstract | 2 | See the PRISMA 2020 for Abstracts checklist. | Abstract |
| **INTRODUCTION** | | |  |
| Rationale | 3 | Describe the rationale for the review in the context of existing knowledge. | Page 2 |
| Objectives | 4 | Provide an explicit statement of the objective(s) or question(s) the review addresses. | Page 3 |
| **METHODS** | | |  |
| Eligibility criteria | 5 | Specify the inclusion and exclusion criteria for the review and how studies were grouped for the syntheses. | Table 1 |
| Information sources | 6 | Specify all databases, registers, websites, organizations, reference lists and other sources searched or consulted to identify studies. Specify the date when each source was last searched or consulted. | Page 3-4 |
| Search strategy | 7 | Present the full search strategies for all databases, registers and websites, including any filters and limits used. | Supplementary |
| Selection process | 8 | Specify the methods used to decide whether a study met the inclusion criteria of the review, including how many reviewers screened each record and each report retrieved, whether they worked independently, and if applicable, details of automation tools used in the process. | Page 4 |
| Data collection process | 9 | Specify the methods used to collect data from reports, including how many reviewers collected data from each report, whether they worked independently, any processes for obtaining or confirming data from study investigators, and if applicable, details of automation tools used in the process. | Page 4 |
| Data items | 10a | List and define all outcomes for which data were sought. Specify whether all results that were compatible with each outcome domain in each study were sought (e.g., for all measures, time points, analyses), and if not, the methods used to decide which results to collect. | - |
|  | 10b | List and define all other variables for which data were sought (e.g., participant and intervention characteristics, funding sources). Describe any assumptions made about any missing or unclear information. | Page 5 |
| Study risk of bias assessment | 11 | Specify the methods used to assess risk of bias in the included studies, including details of the tool(s) used, how many reviewers assessed each study and whether they worked independently, and if applicable, details of automation tools used in the process. | Page 5 |
| Effect measures | 12 | Specify for each outcome the effect measure(s) (e.g., risk ratio, mean difference) used in the synthesis or presentation of results. | - |
| Synthesis methods | 13a | Describe the processes used to decide which studies were eligible for each synthesis (e.g., tabulating the study intervention characteristics and comparing against the planned groups for each synthesis (item #5)). | Page 5-6 |
|  | 13b | Describe any methods required to prepare the data for presentation or synthesis, such as handling of missing summary statistics, or data conversions. | - |
|  | 13c | Describe any methods used to tabulate or visually display results of individual studies and syntheses. | - |
|  | 13d | Describe any methods used to synthesize results and provide a rationale for the choice(s). If meta-analysis was performed, describe the model(s), method(s) to identify the presence and extent of statistical heterogeneity, and software package(s) used. | - |
|  | 13e | Describe any methods used to explore possible causes of heterogeneity among study results (e.g., subgroup analysis, meta-regression). | - |
|  | 13f | Describe any sensitivity analyses conducted to assess robustness of the synthesized results. | - |
| Reporting bias assessment | 14 | Describe any methods used to assess risk of bias due to missing results in a synthesis (arising from reporting biases). | - |
| Certainty assessment | 15 | Describe any method used to assess certainty (or confidence) in the body of evidence for an outcome. | - |
| **RESULTS** | | |  |
| Study selection | 16a | Describe the results of the search and selection process, from the number of records identified in the search to the number of studies included in the review, ideally using a flow diagram. | Figure 1 |
|  | 16b | Cite studies that might appear to meet the inclusion criteria, but which were excluded, and explain why they were excluded. | Figure 1 |
| Study characteristics | 17 | Cite each included study and present its characteristics. | Table 2 & 3 |
| Risk of bias in studies | 18 | Present assessments of risk of bias for each included study. | Table 1 |
| Results of individual studies | 19 | For all outcomes, present, for each study: (a) summary statistics for each group (where appropriate) and (b) an effect estimate and its precision (e.g., confidence/credible interval), ideally using structured tables or plots. | - |
| Results of syntheses | 20a | For each synthesis, briefly summarize the characteristics and risk of bias among contributing studies. | - |
|  | 20b | Present results of all statistical syntheses conducted. If meta-analysis was done, present for each the summary estimate and its precision (e.g., confidence/credible interval) and measures of statistical heterogeneity. If comparing groups, describe the direction of the effect. | - |
|  | 20c | Present results of all investigations of possible causes of heterogeneity among study results. | - |
|  | 20d | Present results of all sensitivity analyses conducted to assess the robustness of the synthesized results. | - |
| Reporting biases | 21 | Present assessments of risk of bias due to missing results (arising from reporting biases) for each synthesis assessed. | - |
| Certainty of evidence | 22 | Present assessments of certainty (or confidence) in the body of evidence for each outcome assessed. | - |
| **DISCUSSION** | | |  |
| Discussion | 23a | Provide a general interpretation of the results in the context of other evidence. | Page 22-25 |
|  | 23b | Discuss any limitations of the evidence included in the review. | Page 26 |
|  | 23c | Discuss any limitations of the review processes used. | Page 26 |
|  | 23d | Discuss implications of the results for practice, policy, and future research. | Page 25-26 |
| **OTHER INFORMATION** | | |  |
| Registration and protocol | 24a | Provide registration information for the review, including register name and registration number, or state that the review was not registered. | CRD42020211324 |
|  | 24b | Indicate where the review protocol can be accessed, or state that a protocol was not prepared. | PROSPERO |
|  | 24c | Describe and explain any amendments to information provided at registration or in the protocol. | - |
| Support | 25 | Describe sources of financial or non-financial support for the review, and the role of the funders or sponsors in the review. | - |
| Competing interests | 26 | Declare any competing interests of review authors. | - |
| Availability of data, code and other materials | 27 | Report which of the following are publicly available and where they can be found: template data collection forms; data extracted from included studies; data used for all analyses; analytic code; any other materials used in the review. | Page 4 |

*From:*  Page, M.J., McKenzie, J.E., Bossuyt, P.M., Boutron, I., Hoffmann, T.C., Mulrow, C.D., … Moher, D. (2021). The PRISMA 2020 statement: An updated guideline for reporting systematic reviews. *BMJ*, *372*, n71. doi: 10.1136/bmj.n71

**The ENTREQ checklist**

| **No** | **Item** | **Guide and description** | **Reported on page #** |
| --- | --- | --- | --- |
| 1 | Aim | State the research question the synthesis addresses. | 3 |
| 2 | Synthesis methodology | Identify the synthesis methodology or theoretical framework that underpins the synthesis and describe the rationale for choice of methodology (e.g., meta-ethnography, thematic synthesis, critical interpretive synthesis, grounded theory synthesis, realist synthesis, meta-aggregation, meta-study, framework synthesis). | 3 |
| 3 | Approach to searching | Indicate whether the search was pre-planned (comprehensive search strategies to seek all available studies) or iterative (to seek all available concepts until theoretical saturation is achieved). | 4 |
| 4 | Inclusion criteria | Specify the inclusion/exclusion criteria (e.g., in terms of population, language, year limits, type of publication, study type). | 4-5 |
| 5 | Data sources | Describe the information sources used (e.g., electronic databases (MEDLINE, EMBASE, CINAHL, psychINFO, Econlit), grey literature databases (digital thesis, policy reports), relevant organizational websites, experts, information specialists, generic web searches (Google Scholar), hand searching, reference lists) and when the searches were conducted; provide the rationale for using the data sources. | 4 |
| 6 | Electronic search strategy | Describe the literature search (e.g., provide electronic search strategies with population terms, clinical or health topic terms, experiential or social phenomena related terms, filters for qualitative research and search limits). | 4 and Supplementary material |
| 7 | Study screening methods | Describe the process of study screening and sifting (e.g., title, abstract and full text review, number of independent reviewers who screened studies). | 4 and Figure 1 |
| 8 | Study characteristics | Present the characteristics of the included studies (e.g., year of publication, country, population, number of participants, data collection, methodology, analysis, research questions). | Table 2 & 3 |
| 9 | Study selection results | Identify the number of studies screened and provide reasons for study exclusion (e.g. for comprehensive searching, provide numbers of studies screened and reasons for exclusion indicated in a figure/flowchart; for iterative searching describe reasons for study exclusion and inclusion based on modifications t the research question and/or contribution to theory development). | 4 and Figure 1,  Supplementary material |
| 10 | Rationale for appraisal | Describe the rationale and approach used to appraise the included studies or selected findings (e.g., assessment of conduct (validity and robustness), assessment of reporting (transparency), assessment of content and utility of the findings). | 4 and Table 1 |
| 11 | Appraisal items | State the tools, frameworks and criteria used to appraise the studies or selected findings (e.g., Existing tools: CASP, QARI, COREQ, Mays and Pope [25]; reviewer developed tools; describe the domains assessed: research team, study design, data analysis and interpretations, reporting). | 4 and Table 1 |
| 12 | Appraisal process | Indicate whether the appraisal was conducted independently by more than one reviewer and if consensus was required. | 5 |
| 13 | Appraisal results | Present results of the quality assessment and indicate which articles, if any, were weighted/excluded based on the assessment and give the rationale. | Table 1 |
| 14 | Data extraction | Indicate which sections of the primary studies were analyzed and how the data were extracted from the primary studies? (e.g., all text under the headings “results /conclusions” were extracted electronically and entered into computer software). | 5-6 |
| 15 | Software | State the computer software used, if any. | n/a |
| 16 | Number of reviewers | Identify who was involved in coding and analysis. | 7 |
| 17 | Coding | Describe the process for coding of data (e.g., line-by-line coding to search for concepts). | 6-7 |
| 18 | Study comparison | Describe how comparisons were made within and across studies (e.g., subsequent studies were coded into pre-existing concepts, and new concepts were created when deemed necessary). | 6 |
| 19 | Derivation of themes | Explain whether the process of deriving the themes or constructs was inductive or deductive. | 7 |
| 20 | Quotations | Provide quotations from the primary studies to illustrate themes/constructs, and identify whether the quotations were participant quotations or the author’s interpretation | 16-22 |
| 21 | Synthesis output | Present rich, compelling and useful results that go beyond a summary of the primary studies (e.g., new interpretation, models of evidence, conceptual models, analytical framework, development of a new theory or construct). | 22-25 |

*From:* Tong, A., Flemming, K., McInnes, E., Oliver, S., & Craig, J. (2012). Enhancing transparency in reporting the synthesis of qualitative research: ENTREQ. *BMC Medical Research Methodology*, *12*(181), 1-8. doi: 10.1186/1471-2288-12-181

**Systematic search strategy**

PubMed

| **Search** | **Query** | **Items found** |
| --- | --- | --- |
| [#1](https://www.ncbi.nlm.nih.gov/pubmed) | Search “Parents”[MeSH Terms] | 88,979 |
| [#2](https://www.ncbi.nlm.nih.gov/pubmed) | Search “Fathers”[MeSH Terms] | 7,367<https://www.ncbi.nlm.nih.gov/pubmed/?cmd=HistorySearch&querykey=2> |
| [#3](https://www.ncbi.nlm.nih.gov/pubmed) | Search “Mothers”[MeSH Terms] | 33,755 |
| [#4](https://www.ncbi.nlm.nih.gov/pubmed) | Search (parent OR parents OR parent's OR parents' OR father OR fathers OR father's OR fathers' OR mother OR mothers OR mother's OR mothers' OR parental OR maternal) | 592,074 |
| [**#**](https://www.ncbi.nlm.nih.gov/pubmed)**5**  **KEY CONCEPT (PARENT)** | **COMBINE #1 OR #2 OR #3 OR #4** | [612,094](https://www.ncbi.nlm.nih.gov/pubmed/?cmd=HistorySearch&querykey=24) |
| [#](https://www.ncbi.nlm.nih.gov/pubmed)6 | Search “Child”[MeSH Terms] | 1,699,690 |
| #7<https://www.ncbi.nlm.nih.gov/pubmed> | Search “Adolescent”[MeSH Terms] | 1,774,862 |
| [#](https://www.ncbi.nlm.nih.gov/pubmed)8 | Search (pediatric OR pediatrics OR pediatric OR pediatrics OR child OR children OR children's OR childhood OR daughter OR daughters OR daughter's OR daughters' OR teen OR teens OR teenager OR teenagers OR girl OR girls OR adolescent OR adolescents OR adolescence OR youth OR youths OR "young person" OR "young persons") | [1,516,524](https://www.ncbi.nlm.nih.gov/pubmed/?cmd=HistorySearch&querykey=9) |
| [**#**](https://www.ncbi.nlm.nih.gov/pubmed)**9**  **KEY CONCEPT (CHILD)** | **COMBINE #6 OR #7 OR #8** | [3,145,284](https://www.ncbi.nlm.nih.gov/pubmed/?cmd=HistorySearch&querykey=25) |
| [#1](https://www.ncbi.nlm.nih.gov/pubmed)0 | Search “Papillomavirus Vaccines”[Majr] | 5,563 |
| [#1](https://www.ncbi.nlm.nih.gov/pubmed)1 | Search ("human papillomavirus" OR "human papillomavirus vaccine" OR "human papillomavirus vaccines" OR "human papillomavirus vaccination" OR "human papillomavirus vaccinations" OR "human papillomavirus immunisation" OR "human papillomavirus immunisations" OR "human papillomavirus immunization" OR "human papillomavirus immunizations" OR HPV) | [38,970](https://www.ncbi.nlm.nih.gov/pubmed/?cmd=HistorySearch&querykey=14) |
| [**#1**](https://www.ncbi.nlm.nih.gov/pubmed)**2**  **KEY CONCEPT (HPV)** | **COMBINE #10 OR #11** | [39,598](https://www.ncbi.nlm.nih.gov/pubmed/?cmd=HistorySearch&querykey=15) |
| [#](https://www.ncbi.nlm.nih.gov/pubmed)13 | Search "Culture"[Mesh] | 141,915 |
| [#1](https://www.ncbi.nlm.nih.gov/pubmed)4 | Search (culture OR cultures OR cultural OR custom OR customs OR tradition OR traditions OR traditional OR ethnic OR ethnics OR ethnicity OR “ethnic group” OR “ethnic groups” OR minor OR minors OR minority OR minorities OR race OR races OR racial OR religion OR religions OR religious OR belief OR beliefs OR background OR backgrounds OR value OR values) | 4,055,577 |
| **#15**  **KEY CONCEPT**  **(CULTURAL PERSPECTIVE)** | **COMBINE #13 OR #14** | 4,128,751 |
| [#](https://www.ncbi.nlm.nih.gov/pubmed)16 | Search “Qualitative Research”[MeSH Terms] | 31,474 |
| [#](https://www.ncbi.nlm.nih.gov/pubmed)17 | Search “Focus Groups”[MeSH Terms] | 21,706 |
| #18 | Search (qualitative OR “qualitative study” OR “qualitative studies” OR “qualitative research” OR “focus group” OR “focus groups” OR descriptive OR description OR explorative OR exploratory OR ethnology OR ethnological OR meta-ethnography OR ethnography OR ethnographic OR “grounded theory” OR phenomenological OR phenomenology OR interview OR interviews OR narrative OR narratology OR “narrative research” OR “thematic analysis”) | [93](https://www.ncbi.nlm.nih.gov/pubmed/?cmd=HistorySearch&querykey=20)6,044 |
| **#19**  **KEY CONCEPT (QUALITATIVE)** | **COMBINE #16 OR #17 OR #18** | 936,067 |
| **#20** | **COMBINE #5 AND #9 AND #12 AND #14 AND #19**  FILTERED English | 217  **214** |
